# Experimental comparative study of arterial implants made of silicone reinforced with polyester fabric and expanded polytetrafluoroethylene (PTFE) in rabbits aorta

**DOI:** 10.1590/0100-6991e-20202587

**Published:** 2021-01-26

**Authors:** FERNANDA APPOLONIO ROCHA, LAILA MASSAD RIBAS, PAULO ISAO SASSAKI-NETO, NELSON DE-LUCCIA

**Affiliations:** 1 - Faculdade de Medicina da Universidade de São Paulo - FMUSP, Universidade de São Paulo, Departamento de Cirurgia - São Paulo - SP - Brasil; 2 - Universidade Federal de Pernambuco - UFPE, Departamento de Cirurgia - CCM - Recife - PE - Brasil

**Keywords:** Blood Vessel Prosthesis, Dimethylpolysiloxanes, Polytetrafluoroethylene, Implants, Experimental, Rabbits, Prótese Vascular, Dimetilpolisiloxanos, Politetrafluoretileno, Implantes Experimentais, Coelhos

## Abstract

**Objectives:**

the aim of this study was to compare the outcomes of a new silicone vascular prostheses with PTFE vascular prostheses, on a rabbit experimental model*.*

**Methods:**

forty rabbits underwent infra-renal aorta replacement with 4 mm diameter prostheses, twenty animals with PDMS and twenty animals with PTFE (control group). Retrograde aortic angiography was performed to assess patency. Histological graft samples were examined by electron microscopy to evaluate prostheses endothelialization*.*

**Results:**

patency rates were 100% for both grafts after 30 days; after 60 days, patency rate for PDMS was 92.3% (±7.4), and 73,8% (±13.1) at 90 days. PTFE grafts had patency rates of 87.5% (±11.7) at 60 and 90 days. No statistically significant difference was found in between groups for patency rates (p=0.62). Postoperative complications (death, paraplegia) rates (p=0.526) and aortic clamping times (p=0.299) were comparable in both groups. No statistically significant difference for stenosis was found on angiographical analysis between groups (p=0.650). Electron microscopy revealed limited anastomotic endothelial ingrowth in both prostheses.

**Conclusion:**

in this experimental model, PDMS and PTFE vascular prostheses had comparable outcomes and PDMS prosthesis could be used as a vascular graft.

## RESUMO

**Objetivo:**

analisar novo tubo de silicone como material para prótese vascular e compará-lo a prótese de PTFE, em modelo experimental com coelhos.

**Métodos:**

quarenta coelhos foram submetidos a interposição, na aorta infrarrenal, de próteses de 4mm de diâmetro, sendo 20 animais com PDMS e 20 com PTFE (grupo controle). Foi realizada arteriografia retrógrada da aorta para avaliar a patência das próteses. Para avaliar a endotelização das próteses foi realizada microscopia eletrônica de maneira amostral pareada.

**Resultados:**

a patência em 30 dias foi de 100% para as duas próteses. Aos 60 dias, a taxa de patência do PDMS foi de 92,3% (± 7,4), e de 73,8% (±13,1) em 90 dias; as próteses de PTFE tiveram taxas de patência de 87,5% (± 11,7) aos 60 e 90 dias. Não foi observada diferença significativa entre as taxas de patência dos grupos (p=0,62). Não foi observada diferença entre os grupos quanto à evolução com complicações pós-operatórias (p=0,526) e quanto ao tempo de clampeamento da aorta (p=0,299). Não houve diferença significativa entre os grupos quanto ao grau de estenose das próteses (p=0,650) à avaliação angiográfica. A microscopia eletrônica mostrou crescimento endotelial limitado às regiões próximas às anastomoses nos dois tipos de próteses.

**Conclusões:**

o PDMS mostrou-se passível de utilização como prótese vascular, com resultados comparáveis aos do PTFE no modelo utilizado.

## INTRODUCTION

The use of synthetic grafts for large caliber arteries has been done for years and with broad support from medical literature. In arteries such as the aorta, iliac, and femoral arteries, high flow and low resistance guarantee long-lasting patency of vascular prostheses of dacron or polytetrafluoroethylene (PTFE)^1^ ^-^ ^5^. However, in small-caliber vessels, none of these materials was found to be superior or equal to the saphenous vein^6^ ^-^ ^9^ ^,^ considered the arterial substitute of choice for peripheral revascularization^10^ ^,^ ^11^. 

In cases where there is no autologous vascular segment with sufficient diameter and extension to be used in bypass surgery, especially in distal revascularizations of the lower limb, it is necessary to resort to synthetic grafts, despite their known inferiority^11^. 

Several studies have been carried out in this field in an attempt to find a substitute comparable to the autologous vein, such as cryopreserved veins^12^ ^,^ ^13^ and biosynthetic materials^14^ ^,^ ^15^. However, an effective alternative to replace small vessels is still being sought^16^ ^,^ ^17^. 

Polydimethylsiloxane (PDMS), or silicone, has been used in medicine since the 1960s^18^ ^,^ ^19^. Due to its characteristics, silicone has become one of the most used materials for prosthetic replacement in various contexts, such as breast and penile prostheses. The use of PDMS in the manufacture of various types of catheters for intravenous administration of substances is also widely and universally accepted. It is an inert material, which is one of the advantages for use in implants. Silicone also has excellent long-term bio-stability, low toxicity, and low thrombogenicity^20^ ^-^ ^26^. 

De Luccia and De Luccia^27^ developed a small caliber vascular prosthesis (internal diameter of 4 mm), of medical grade silicone in the inner (luminal) and outer layers, with reinforcement of polyester fabric as an intermediate layer, in order to increase resistance to tearing and preventing expansion. 

The hypothesis that PDMS prostheses can be used as substitutes for small caliber arteries represents the reason for the present research. The aim of the study was to compare, in an experimental model for small vessels, the PDMS prosthesis with the PTFE one, frequently used in arterial revascularizations.

## METHODS

All surgical procedures were performed on domestic rabbits (Oryctolagus cuniculus) at the Department of Surgery, Faculty of Medicine, University of São Paulo. The animals were supplied by the vivarium of that institution and were treated in accordance with the principles established by the Animal Welfare Act and the NIH Guide for the Care and Use of Laboratory Animals. This study was carried out with the approval of the ethics committee of the Hospital das Clínicas of the Faculty of Medicine of the University of São Paulo (no. 0716/09). 

We included 40 animals in the study, which we divided into two groups, according to the implanted prosthesis: PDMS group (rabbits submitted to PDMS prosthesis implantation; n = 20) and PTFE group (rabbits submitted to PTFE prosthesis implantation; n = 20). 

### Prostheses

**Figure 1:**
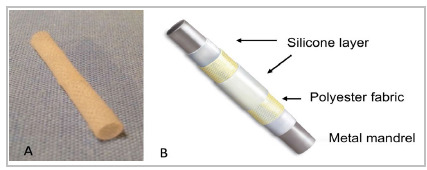
PDMS prosthesis. A - PDMS tubular prosthesis (0.4 mm wall and 4 mm diameter); B - Illustrative diagram of the PDMS prosthesis layers.

PDMS prostheses were manufactured according to the patent of De Luccia and De Luccia^27^. The medical grade silicone in liquid form was mixed with a curing agent and applied over a metal mandrel covered with polyester fabric to increase the tear resistance of the silicone. To obtain uniform curing, the rotational movement of the mandrel was maintained, initially at room temperature and then in an oven to reach a post-cure temperature of 110^o^C for 30 minutes. The PDMS prosthesis had a 0.4 mm tubular wall and an internal diameter of 4 mm. The internal and external surfaces were composed of silicone. The polyester fabric was placed as an intermediate layer on the wall in order to reinforce it (Figure 1). The PTFE prostheses used were tubular, straight, thin-walled (0.41 mm), and without external annular reinforcement, with a diameter of 4 mm.

### Anesthetic technique

The animals were anesthetized with a intramuscular injection of 10% ketamine hydrochloride (35 mg/kg, Ketalar^®^; Cristalia, São Paulo, Brazil) and 2% xylazine hydrochloride (5 mg/kg, Rompum^®^; Bayer AG, Leverkusen, Germany). During surgical procedures, the animals received saline solution (0.9% sodium chloride) through a cannulated 22G catheter in the marginal ear vein.

### Surgical technique

**Figure 2:**
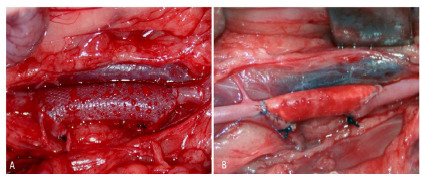
Prostheses implanted in the experimental animals. A- PDMS prosthesis; B- PTFE prosthesis.

We performed a median laparotomy, with transperitoneal approach, which allowed the dissection of 3 to 4 cm of the infrarenal aorta. We used a self-static retractor, developed specifically for the research, to prevent evisceration during the exposure. We carefully preserved the lumbar arteries. Before aortic clamping with microsurgical forceps, we administered sodium heparin (200 IU / kg iv; Hepamax^®^, Blausiegel, São Paulo, Brazil). We performed anastomoses of the prosthesis (PDMS or PTFE) to the aorta by an termino-lateral technique, with running sutures of 7-0 polypropylene, with a 3/8 - 1 cm cardiovascular needle (Proleneâ, Ethicon). The proximal anastomosis was the first to be performed and, after its completion, the clamps were released for a few minutes in order to restore perfusion to the lower limbs and provide tissue protection by ischemic preconditioning. Then, we replaced the clamps and made the distal anastomosis. After the anastomoses were completed, the clamps were removed and the aorta was ligated between the anastomoses on two levels, with 4-0 cotton suture, followed by the section of the vessel between the two ligatures (Figure 2). At the end of the procedure, the animals received postoperative analgesia with 2% Meloxican (Maxicam^®^, Ouro Fino), at a dose of 0.2 mg/kg, intramuscularly.

### Prosthesis evaluations

After a minimum period of 28 days, the prostheses were evaluated by aortic fluoroscopy. The images were acquired on a Diasonics OECs 9000 fluoroscopy scanner (Salt Lake City, Utah, USA). Dissection of the femoral artery was performed by unilateral inguinal incision to inject the contrast agent diatrizoate meglumine (Reliev 60%; BerliMed SA, São Paulo, Brazil). Through aortography, we determined the flow and degree of stenosis of the prostheses. At the end of the experimental procedures, the animals were anesthetized and euthanized with 19.1% potassium chloride (Isofarma, Eusébio, CE, Brazil), and the corpses were discarded according to the routine procedures of the surgical department.

The prostheses were removed after euthanasia, and samples were sent to scanning electron microscopy; the images were obtained using the Philips XL30 system (FEI, Hillsboro, Oregon, USA). Optical microscopy was not possible due to the characteristics of the material, which was explanted by the microtome when attempting to prepare the slides.

### Statistical analysis

To analyze the prostheses’ flow and the risk of occlusion (measured in days) we used the Kaplan-Meier non-parametric estimator, and to compare the flow curves, we used the logrank test. Occlusion of the prosthesis identified in the catheterization was considered as an event. The animals that had a viable prosthesis at the time of the aortography and that were euthanized after the exam where censored on the exam date. The hypothesis of equality of means was rejected by a p < 0.05 (5%).

## RESULTS

We performed the procedures on 40 animals, 20 in each group.

Twenty-five animals, 14 from the PDMS group and 11 from the PTFE group, survived until late evaluations. All surviving animals underwent angiographic control at the end of the observation period and were analyzed using Kaplan-Meier curves.

We observed early mortality (up to two days after the procedure) in eight animals (three in the PDMS group and five in the PTFE group). Seven animals developed paraplegia in the immediate postoperative period (three from the PDMS group and four from the PTFE group) and were euthanized. These animals were not considered in the long-term follow-up.

There was no difference between the groups regarding postoperative complications (death and paraplegia - Fisher’s exact test, p = 0.526).

### Surgical characteristics

The walls of the PDMS prosthesis had characteristics of flexibility, complacency and ease of handling, which allowed easy passage of the needle and retention and containment of the suture lines. In addition, the prosthesis provided for easily palpable pulse. We identified the described characteristics in all operations. We achieved satisfactory hemostasis at the end of the experiments and the presence of a pulse distal to the anastomoses attested to the immediate patent flow of the prostheses in all animals. The placement of clamps on the PDMS prosthesis did not cause any deformity, and after removing them, the prosthesis resumed the tubular shape.

The PTFE prosthesis has little elasticity and more rigid walls, which made the needle more difficult to penetrate and the suture was more laborious. In addition, this type of prosthesis did not integrate to the native artery as well as the PDMS prostheses due to the rigidity of the walls. Another characteristic observed was the frequent bleeding through the needle holes in the PTFE, which did not occur in PDMS prostheses. The placement of clamps in this type of prosthesis caused the walls to be marked and remain with some initial deformation, but that can be corrected manually.

### Prosthesis evaluation

We followed the animals in the PDMS group for an average of 72 days, with a maximum of 108 days. The animals in the PTFE group were followed for an average of 66 days, with a maximum of 90 days. 

**Figure 3:**
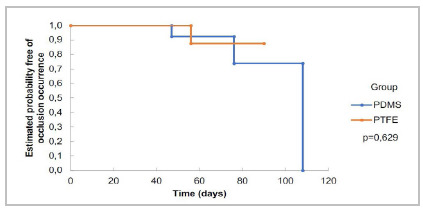
Patency curves (Kaplan-Meier). Proportion of animals with grafts patent in each group. The patency rates for the PDMS (n = 15) were 100% after 30 days, 92.3% after 60 days, and 73.8% after 90 days. In the PTFE group (n = 11), the patency in 30 days was 100%, and in 60 and 90 days it was 87.5%. Logrank test, p = 0.629.

**Figure 4:**
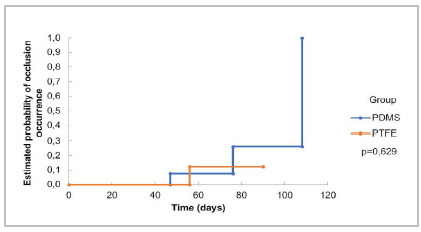
Risk of prosthetic occlusion. Estimated occlusion risk curves, according to the study group.

We measured prosthetic flow in days and evaluated it by aortography exams. The flow rates of the PDMS group (n = 14) were 100% after 30 days, 92.3% (± 7.4) after 60 days, and 73.8% (± 13.1) in 90 days. In the PTFE group (n = 11), the flow in 30 days was 100%, and in 60 and 90 days it was 87.5% (± 11.7). The logrank test showed no statistically significant difference between the groups’ flow rates (p = 0.629) (Figure 3). The risk of occlusion for each group can be seen in Figure 4. 

We observed no hemodynamically significant stenosis in any animal in both groups.

### Macroscopic analysis

The two types of prostheses were surrounded by fibrous scar tissue externally. In all animals receiving implants of PTFE prostheses, they were more adhered, with thicker incorporation tissue, resulting in greater difficulty in the cleavage plane of identification with the tube and removing it for microscopic analysis. All PDMS prostheses showed a more discreet tissue reaction, with more delicate tissue incorporation, which allowed the removal of the specimen to be performed more easily, since the cleavage plane was easily identified and dissected.

There was no case of infection or formation of pseudoaneurysm in the suture lines in either group. PDMS prostheses had no aneurysmatic dilation.

Within the occluded grafts, we observed whitish thrombi, which were probably caused by intimal hyperplasia, later confirmed by scanning electron microscopy.

In prostheses with viable flow, in the internal region the suture lines were covered with a shiny tissue, continuous to the endothelium of the native artery.

### Scanning electron microscopy

**Figure 5:**
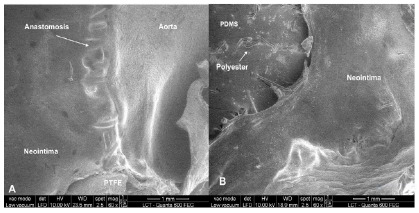
Electron microscopy. Electron microscopy showing the growth of the endothelium (neointima) from the native vessel towards the prostheses A - PTFE; B - PDMS.

We used electron microscopy to provide additional information. We observed endothelial growth of the native vessel towards the prosthetic grafts, covering the suture lines, characterizing the formation of a neointima layer. This formed layer covered the region of the anastomoses and grew just a few millimeters further, with no complete endothelization of the internal surface in any of the prostheses. In some cases, we found more exuberant endothelial growth, corresponding to intimal hyperplasia (Figure 5). 

## DISCUSSION

Vascular prostheses have been used for several decades to restore blood flow in different arterial segments. Although the use of synthetic grafts has been well established for vessels of large caliber, their use is quite limited in vessels with a diameter of less than four millimeters^8^.

In view of the current scenario of synthetic prostheses for small-caliber vessels, a new vascular prosthesis^27^ was developed in PDMS - known as silicone - with polyester fabric reinforcement. Silicone was chosen because it is a material widely used in the medical field and because it has several characteristics considered important for a vascular substitute, such as low toxicity, low thrombogenicity, biocompatibility, and biostability^21^ ^,^ ^23^ ^-^ ^26^ ^,^ ^28^. Reinforcement with polyester fabric aims at increasing silicone grafts’ walls resistance, preventing the occurrence of aneurysmatic dilation and tears. Because it is the most commonly used synthetic graft for revascularization of small-caliber arteries in revascularizations below the knee, we chose PTFE for the control group^7,9 11^.

Experimental models in rabbits are considered good models for evaluating small-caliber vessels, with a diameter between 1 and 4 mm^29^ ^-^ ^32^. The vascular physiology of rabbits is similar in many ways to that of humans, but there are some disadvantages, such as the high rate of postoperative complications (mortality and paraplegia), data compatible with the findings of the study^29^ ^-^ ^32^.

The in vivo experimental model in rabbits demonstrated that the small-caliber PDMS vascular prosthesis was comparable to PTFE in terms of surgical properties, with easy handling and suturing, and without any case of aneurysmatic dilation or infection. 

The flow rate of PDMS grafts (100% after 30 days, 92.3% after 60 days, and 73.8% in 90 days) was similar to that of PTFE grafts (100% in 30 days and 87.5% in 60 and 90 days) in the 25 animals that were included in this analysis. The flow rates of PTFE grafts were similar to those described by Nordestagaard et al.^33^, who carried out a similar study using PTFE prostheses with a diameter of three millimeters also implanted in rabbit aortas. These authors reported a flow rate of 82% after 90 days. 

Intimal hyperplasia is the result of the healing process after vascular injury, and occurs especially in the anastomotic regions, being one of the main causes of stenosis and occlusion of vascular grafts^32^. Intimal hyperplasia in the the suture lines region is more frequent with the use of synthetic prostheses than with venous grafts.

In the prostheses evaluations, the flow was demonstrated by injection of contrast into the aorta (using arteriography performed by retrograde catheterization) and long-term monitoring. 

The presence of neointima covering only the anastomosis site in the suture line, detected by electron microscopy in both prostheses is also observed in humans, even many years after the implant^31^ ^,^ ^34^.

Some limitations of the study deserve mention. The duration of the study is considered to be short-term when assessing flow and intimal hyperplasia. In addition, the model used displayed a high mortality rate and only 25 animals were included for flow analysis, which may limit our ability to extrapolate the data to actual clinical conditions. However, this brief assessment produces important information about the interaction of the prosthesis with the receptor site. The length of the vascular grafts used is significantly shorter than the length of the bypasses routinely used in humans in cases of peripheral arterial revascularizations. Despite the mentioned limitations, the model used seems to be valid for small-caliber vascular grafts^29^ ^-^ ^32^.

Studies with long-term follow-up, using longer grafts, and in other animal models are necessary in order to evaluate the potential of PDMS as an alternative for small vessels.

## CONCLUSION

In the experimental model used, the PDMS and PTFE vascular prostheses were comparable in terms of short-term flow and endothelialization. PDMS prostheses have good surgical properties.
